# Anti-Hyperglycemic Properties of Crude Extract and Triterpenes from *Poria cocos*

**DOI:** 10.1155/2011/128402

**Published:** 2010-09-16

**Authors:** Tzu-Hsuan Li, Chia-Chung Hou, Cicero Lee-Tian Chang, Wen-Chin Yang

**Affiliations:** ^1^Agricultural Biotechnology Research Center, Academia Sinica, Taipei 115, Taiwan; ^2^Department and Institute of Pharmacology, National Yang-Ming University, Taipei 112, Taiwan; ^3^Department of Veterinary Medicine, National Chung Hsing University, Taichung, Taiwan; ^4^Institute of Zoology, National Taiwan University, Taipei 10617, Taiwan; ^5^Department of Life Sciences, National Chung Hsing University, Taichung, Taiwan

## Abstract

*Poria cocos*, Bai Fu Ling in Chinese, is used in traditional Chinese medicine to treat diabetes. However, its claimed benefits and mechanism are not fully understood. This study aimed to investigate the effect and action of *P. cocos* on type 2 diabetes. We first performed phytochemical analysis on the crude extract and factions of *P. cocos*. *P. cocos* crude extract at 50 mg/kg body weight or more significantly decreased blood glucose levels in db/db mice. Based on a bioactivity-directed fractionation and isolation (BDFI) strategy, chloroform fraction and subfractions 4 and 6 of the *P. cocos* crude extract possessed a blood glucose-lowering effect. Dehydrotumulosic acid, dehydrotrametenolic acid, and pachymic acid were identified from the chloroform sub-fractions 4, 3, and 2, respectively. Dehydrotumulosic acid had anti-hyperglycemic effect to a greater extent than dehydrotrametenolic acid and pachymic acid. Mechanistic study on streptozocin- (STZ-) treated mice showed that the crude extract, dehydrotumulosic acid, dehydrotrametenolic acid, and pachymic acid of *P. cocos* exhibited different levels of insulin sensitizer activity. However, the *P. cocos* crude extract and triterpenes appeared not to activate PPAR-*γ* pathway. Overall, the data suggest that the *P. cocos* extract and its triterpenes reduce postprandial blood glucose levels in db/db mice via enhanced insulin sensitivity irrespective of PPAR-*γ*.

## 1. Introduction

Diabetes mellitus is a life-threatening chronic metabolic disease which currently afflicts 3% of the world population. Over 90% of diabetic populations are diagnosed with type 2 diabetes [1, 2]. Diabetes is caused by a defect in insulin production, insulin action, or both [3]. The defect impairs glucose homeostasis in diabetic patients, resulting in hyperglycemia, a hallmark of diabetes [4]. Therefore, one important method of treating patients with type 2 diabetes is to control blood glucose levels, which can be achieved by an increase of insulin release (insulin releasers) or insulin action (insulin sensitizers), a decrease of intestinal glucose uptake (*α*-glucosidase inhibitors), and so forth [5]. However, few clinical drugs are available for diabetes, and those that are available usually have adverse side effects such as decreased efficacy over time and low cost-effectiveness [6–8]. Therefore, research and development of novel drugs for diabetes have been in great demand.

*P. cocos* (Polyporaceae) is a rotten pine-tree fungus. It has long been used as traditional Chinese medicine and food [9–12]. *P. cocos* alone or in combination with other herbs is often used to treat diabetes as well as other disorders [13–15]. Several triterpenes, pachyman, and pachymaran have been identified from *P. cocos* [16–19]. Dehydrotrametenolic acid, one triterpene constituent of *P. cocos*, was shown to reduce hyperglycemia in db/db mice [20]. Besides, dehydrotrametenolic acid was shown to activate peroxide proliferator-activated receptor-*γ* (PPAR-*γ*), a regulator of glucose metabolism, in the same way as the insulin sensitizer ciglitazone [20]. However, this antidiabetic effect and the likely mechanism of *P. cocos* are still poorly studied. 

In this study, we used db/db mice to evaluate the glucose-lowering effects of the crude extract, fractions, and triterpenes of *P. cocos*. Based on a bioactivity-directed fractionation and isolation (BDFI) procedure, triterpenes from the crude extract of *P. cocos* were identified as active compounds. Chromatography and spectroscopy were used to characterize the phytochemistry of *P. cocos*. In addition, STZ-treated mice were used to study the likely mechanism of *P. cocos* and its active triterpenes.

## 2. Materials and Methods

### 2.1. Chemicals, Cells, and Plasmids

Dimethyl sulfoxide (DMSO), glimepiride (GLM), metformin (Met), methanol, ethyl acetate (EA), chloroform, acetonitrile (ACN), trifluoroacetic acid (TFA), and streptozocin (STZ) were purchased from Sigma-Aldrich (MO, USA). Pachymic acid and insulin were purchased from Apin Chemicals Limited (Oxon, UK) and Novo Nordisk (NJ, USA), respectively. 3T3-L1 adipocytes were grown in DMEM medium (Invitrogen, CA, USA) supplemented with 10% bovine calf serum (BCS), 100 units/mL penicillin, 100 *μ*g/mL streptomycin, 6 mM HEPES (Invitrogen, CA, USA), and 2 mM L-glutamine (Invitrogen) at 37°C in 5% CO_2_. pSGGAL-PPAR-*γ*, which expresses a chimeric protein of Gal4 DNA-binding domain linked to PPAR-*γ* ligand-binding domain, and (UAS)5-tk-LUC reporter construct, which contains a pentameric yeast upstream activatory sequence linked to luciferase gene, were generous gifts from Dr. Krister Bamberg (Astra Zeneca, Mölndal, Sweden).

### 2.2. Preparation of Crude Extract, Fractions, and Triterpenes of *P. cocos*

*P. cocos* was purchased from a local market in Taipei, Taiwan. It was authenticated by the Development Center for Biotechnology, Taiwan. Voucher specimens were deposited at Agricultural Biotechnology Research Center. To prepare methanol crude extract of *P. cocos*, dried *P. cocos* was ground into powder. *P. cocos* powder (5 kg) was extracted with methanol (1 : 10 w/v) for 6 days three-times, yielding 7.5‰ methanol extract. The methanol crude extract was dissolved in water (1 L) and partitioned with chloroform (1 L × 5). After obtaining the chloroform fraction (0.82‰), the rest of the extract was further partitioned with ethyl acetate (1 L × 5), yielding an ethyl acetate fraction (2.9‰) and a water fraction (1.8‰). The active chloroform fraction was further fractioned into chloroform sub-fractions 1 (0.10‰), 2 (0.11‰), 3 (0.11‰), 4 (0.12‰), 5 (0.10‰), and 6 (0.24‰) using a normal phase high-performance liquid chromatography (HPLC) column (Phenomenex, Luna 5 *μ* silica, 250 × 10 mm) at a flow rate of 5 ml/min, detected with UV 242 nm. The solvent gradient for HPLC is ethyl acetate (a) and hexane (b): 0 to 60 min: 30% A; 60 to 90 min: 100% A. The 6 chloroform sub-fractions were chemically characterized as published elsewhere [21] using an RP-C18 HPLC column (Phenomenex, Luna 5 *μ* C18, 250 × 4.6 mm) at a flow rate of 1 ml/min, detected with UV 242 nm. The solvent gradient for HPLC is water/0.05% TFA (c) and acetonitrile/0.05% TFA (d): 0 to 15 min: 5 to 15% D; 15 to 35 min: 15 to 20% D; 35 to 65 min: 20 to 40% D; 65 to 100 min: 40 to 100% D; 100 to 110 min: 100% D. Triterpenes were identified from the chloroform sub-fractions using an RP HPLC column (Cosmosil, 5 *μ* C18, 250 × 10 mm) at a flow rate of 5 ml/min, detected with UV 242 nm. The solvent gradient for HPLC was water/0.05% TFA (e) and acetonitrile/0.05% TFA (f): 0 to 10 min: 30 to 52% F; 10 to 50 min: 52 to 100% F; 50 to 60 min: 100% F. Dehydrotumulosic acid, dehydrotrametenolic acid, and pachymic acid were identified by comparing the data obtained from NMR and MS with those published previously in [21–23].

### 2.3. Animal and Drug Administration

Male C57BL/KsJ-db/db mice, which possess a point mutation of the leptin receptor, and male C57BL/6J mice were purchased from the Jackson Laboratory (ME, USA) and the National Laboratory Animal Center (Taipei, Taiwan), respectively. All animals were housed and handled according to the guidelines of the Academia Sinica Institutional Animal Care and Utilization Committee. 

Diabetic C57BL/KsJ-db/db mice aged between 6 to 8 weeks were denied food for 12 h before the experiment and then given free access to food and water for 2 h. On time 0, food was removed (water accessible) and blood sampling from this time point on was considered to be postprandial. After grouping, mice were tube-fed with vehicle, metformin, crude extract, fractions, sub-fractions, or compounds. Postprandial blood glucose level was monitored for 4 h.

C57BL/6J mice aged 5 weeks were treated with an intraperitoneal injection of STZ (200 mg/kg). Mice with postprandial blood glucose level over 500 mg/dL and serum insulin level below 0.18 ng/ml were grouped and tube-fed with control, metformin, glimepiride, crude extract, and compounds (time−1 h). Sixty minutes after tube-feeding of mice, the mice were intraperitoneal injected with insulin at 2.5 IU/kg BW (time 0). Blood glucose levels were measured for 4 h, and all the time points were given free access to food and water. Blood glucose concentration was measured using an Elite glucometer (Bayer, PA, USA). This experiment was modified by previous studies [24, 25].

### 2.4. PPAR-*γ* Reporter Assay

3T3-L1 (10^7^) cells were electroporated with pSGGAL-PPAR-*γ* and (UAS)5-tk-LUC at 260 V and 975 *μ*F using a Bio-Rad electroporator (Bio-Rad, Hercules, CA). Two hours after transfection, cells were divided and treated with DMSO, rosiglitazone (RSG), the crude extract, and triterpenes of *P. cocos *for 24 h. Ten *μ*g of total lysates underwent dual luciferase assays as published in [26]. PPAR-*γ* activity in folds was defined as the firefly/*Renilla *luciferase ratio normalized to the control firefly/*Renilla *luciferase ratio.

### 2.5. Statistical Analysis

The results from three or more independent experiments were presented as mean ± S.E. Data were analyzed by ANOVA. Bonferroni's method was used for post hoc comparisons when appropriate to determine the source of significant differences. Differences of *P*-value less than .05 were considered statistically significant.

## 3. Results

### 3.1. Phytochemical Analysis of Crude Extract, Fractions, Sub-Fractions, and Triterpenes of *P. cocos*

**Figure 1:**
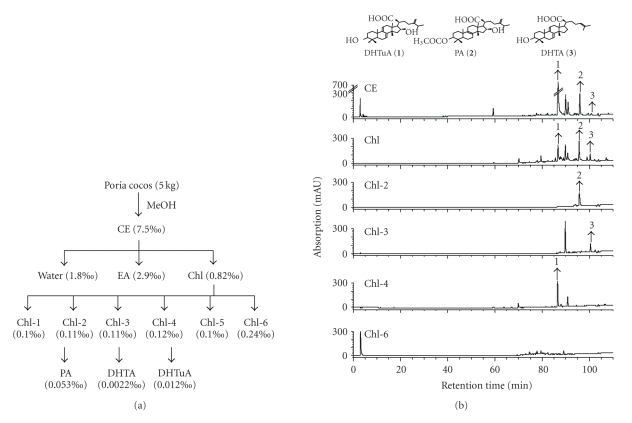
Isolation procedure and HPLC profiles of crude extract, fractions, sub-fractions, and triterpenes of *P. cocos*. (a) Flow chart of preparation procedure of the crude extract, fractions, sub-fractions, and triterpenes of *P. cocos*. The yield of extract, fractions, sub-fractions, and triterpenes is indicated in parentheses. (b) Chemical profiles of crude extract and fractions of *P. cocos* were obtained using HPLC columns and detected with UV detectors. Dehydrotumulosic acid (DHTuA: 1), pachymic acid (PA: 2), and dehydrotrametenolic acid (DHTA: 3) were identified from the Chl-4, Chl-2, and Chl-3 sub-fractions.

*P. cocos* has been used *per se* or together with other herbs as Chinese medicines for diabetes [13]. However, its anti-diabetic effect is poorly understood. In this study, we first chemically characterized crude extract, fractions, and triterpenes of *P. cocos* based on a BDFI procedure (Figure 1(a)). To prepare the crude extract of *P. cocos*, its sclerotia were ground and extracted by methanol. The methanol crude extract was further partitioned with water, ethyl acetate, and chloroform. The active chloroform fraction was separated into 6 sub-fractions. The chloroform sub-fractions 4 and 6 were active and one triterpenes, dehydrotumulosic acid, was identified from the active chloroform sub-fraction 4. Another two triterpenes, pachymic acid and dehydrotrametenolic acid, were isolated from the inactive chloroform sub-fractions 2 and 3. The yield of the crude extract, fractions, sub-fractions, and triterpenes of *P. cocos* is indicated in Figure 1(a). Their HPLC profiles were determined as shown in Figure 1(b).

### 3.2. Glucose-Lowering Effects of Crude Extract, Fractions, Chloroform Sub-Fractions, and Triterpenes of *P. cocos* in db/db Mice

**Figure 2:**
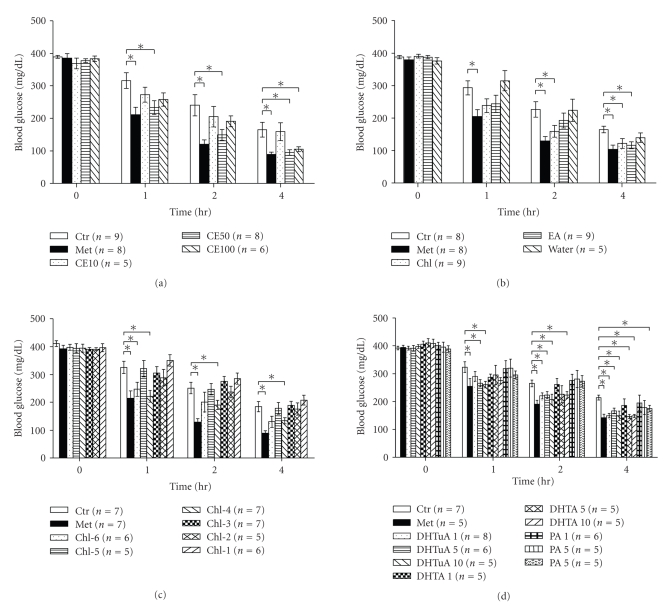
Single-dose effects of crude extract, fractions, sub-fractions, and triterpenes of *P. cocos* on the level of postprandial blood glucose in db/db mice. (a) Diabetic db/db mice were tube-fed with water (Ctr), metformin (Met: 60 mg/kg BW), and crude extract (CE: 10, 50 or 100 mg/kg BW). Blood glucose levels were measured before (0 h) and after (1 to 4 h) tube feeding. (b) The same procedure as (a) except that water (Ctr), metformin (Met: 60 mg/kg BW), water fraction (Water: 50 mg/kg BW), ethyl acetate fraction (EA: 50 mg/kg BW), and chloroform fraction (Chl, 50 mg/kg BW) were administered. (c) The same procedure as (a) except that water (Ctr), metformin (Met: 60 mg/kg BW), and 6 chloroform sub-fractions (Chl-1 to Chl-6: 50 mg/kg BW) were administered. (d), The same procedure as (a) except that DMSO/water (Ctr), metformin (Met: 60 mg/kg), dehydrotumulosic acid (DHTuA: 1, 5, and 10 mg/kg BW), pachymic acid (PA: 1, 5, and 10 mg/kg BW), and dehydrotrametenolic acid (DHTA: 1, 5, and 10 mg/kg BW) were administered. All the above data are expressed as mean ± S.E. *P; *(*) less than .05 is considered to be statistically significant. The mouse number (*n*) is indicated in parenthesis.

Next, a mouse model of type 2 diabetes, db/db mice, was used to evaluate the anti-diabetic potential of *P. cocos*. First, we examined the glucose-lowering effect of crude extract of *P. cocos* in db/db mice. Diabetic db/db mice aged 6 to 8 weeks spontaneously developed hyperglycemia, and their postprandial blood glucose levels were somewhere between 350 and 400 mg/dL (Figure 2(a)). In contrast, the levels of blood glucose in db/db mice receiving a dose of control vehicle dropped to 165 mg/dL over 4 h (Figure 2(a)). As expected, metformin significantly lowered the levels of blood glucose (Figure 2(a)) in db/db mice. Crude extract of *P. cocos* at a single dose of 50 or 100 mg/kg body weight also significantly reduced the blood glucose levels (Figure 2(a)).

Next, we tested the glucose-lowering effect of the water, ethyl acetate, and chloroform fractions of *P. cocos* on db/db mice. The levels of blood glucose in mice receiving a dose of control vehicle decreased to 164.5 mg/dL over 4 h (Figure 2(b)). Metformin effectively decreased the blood glucose levels (Figure 2(b)). In contrast, the chloroform fraction, but not the water and ethyl acetate fractions, significantly lowered blood glucose levels (Figure 2(b)). Next, we further evaluated the glucose-lowering effect of chloroform sub-fractions 1–6, resulting from the active *P. cocos* chloroform fraction. One dose of metformin effectively decreased the blood glucose levels in db/db mice as compared to control vehicle. Chloroform sub-fractions 4 and 6 significantly lowered the blood glucose levels to a greater extent than the other 4 sub-fractions (Figure 2(c)). This lowering effect of sub-fraction 4 (50 mg/kg body weight) seemed to last longer than that of sub-fraction 6 (50 mg/kg body weight) (Figure 2(c)). 

Finally, we evaluated the glucose-lowering effects of triterpenes in db/db mice. Like metformin, dehydrotumulosic acid showed a significant glucose-lowering activity in db/db mice (Figure 2(d)). In contrast, dehydrotrametenolic acid and pachymic acid, isolated from the less active chloroform sub-fraction 3 and 2, showed lower glucose-lowering activities. However, we failed to identify active compounds from the active chloroform sub-fraction 6 of *P. cocos*.

### 3.3. Mechanistic Study of the Crude Extract and Triterpenes of *P. cocos*

**Figure 3:**
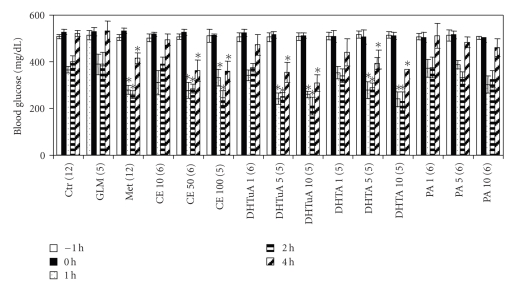
Glucose-lowering effects of the crude extract of *P. cocos* and triterpenes in STZ-treated mice. C57BL mice, which have received a high dose of STZ, were tube-fed with water (Ctr), glimepiride (GLM: 2.5 mg/kg BW), metformin (Met: 60 mg/kg BW), crude extract (CE: 10, 50, or 100 mg/kg BW), dehydrotumulosic acid (DHTuA: 1, 5, or 10 mg/kg BW), dehydrotrametenolic acid (DHTA: 1, 5, and 10 mg/kg BW), and pachymic acid (PA: 1, 5, or 10 mg/kg). Blood glucose levels were measured before (−1 and 0 h) and after (1 to 4 h) tube feeding and injection of insulin 2.5 IU/kg BW. All the above data are expressed as mean ± S.E. *P; *(*) indicates the difference between control (Ctr) and experimental group. The mouse number (*n*) is indicated in parenthesis.

Above we have shown that like metformin, *P. cocos* and its triterpenes have different hypoglycemic effects in db/db mice. To understand the anti-diabetic mechanism of the crude extract of *P. cocos* and triterpenes, we examined their glucose-lowering effects in STZ-treated mice whose *β* islet cells were destroyed. Insulin administration was able to decrease blood glucose levels in STZ mice (Figure 3). As expected, glimepiride, an insulin releaser, had no effect on insulin-mediated blood glucose reduction in STZ-treated mice (Figure 3). In contrast, metformin, an insulin sensitizer, effectively augmented the insulin-mediated blood glucose reduction (Figure 3). In the same way as metformin, the crude extract of *P. cocos* also augmented the insulin-mediated blood glucose reduction (Figure 3). Similar results were obtained for dehydrotumulosic acid, dehydrotrametenolic acid and, if any, pachymic acid (Figure 3). These results imply that *P. cocos* and triterpenes have the activities of insulin sensitizer but not releaser.

**Figure 4:**
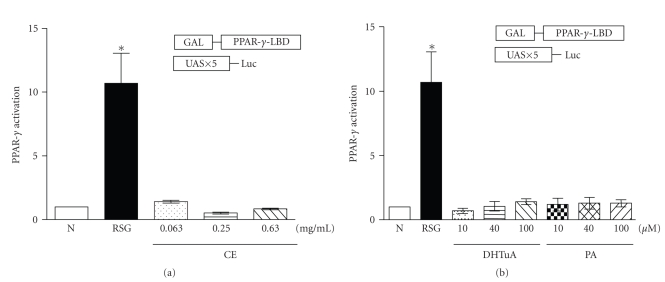
Effects of the *P. cocos* crude extract, dehydrotumulosic acid (DHTuA), and pachymic acid (PA) on PPAR-*γ* activation. (a) 3T3-L1 cells were transiently transfected with reporter constructs, pSGGAL4-PPAR-*γ* (2 *μ*g), and (UAS)5-tk-LUC plasmid (10 *μ*g). The cells were treated with DMSO (N: negative control), rosiglitazone (RSG: 1 *μ*M), and the *P. cocos* crude extract (0.063, 0.25, and 0.63 mg/ml). After 24 h, cell lysates were subjected to dual luciferase assay. (b) The same procedure as (a) except that the cells were treated with dehydrotumulosic acid (DHTuA: 10, 40, or 100 *μ*M) and pachymic acid (PA: 10, 40, or 100 *μ*M). All the data in folds are expressed as mean ± S.E. *P;* (*) less than .05 is considered to be statistically significant.

**Figure 5:**
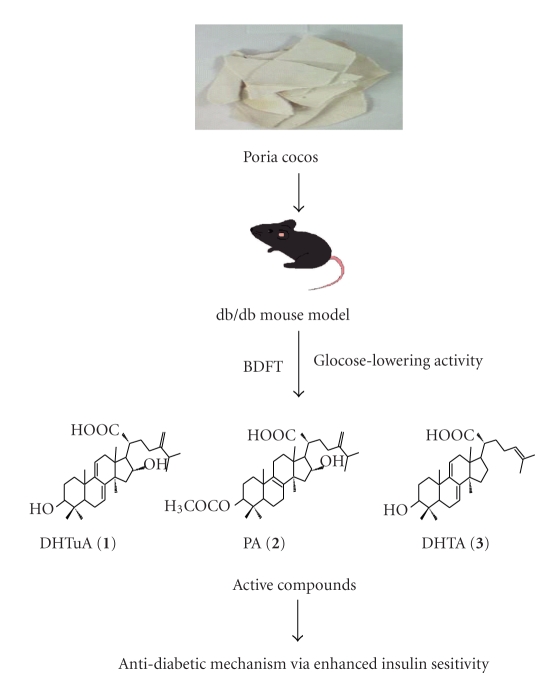
BDFI approach to study* P. cocos* and its active compounds for diabetes.

PPAR-*γ* plays a role in glucose metabolism and is a target of insulin sensitizing drugs. Therefore, we further examined whether the crude extract of *P. cocos* and triterpenes regulated PPAR-*γ* activation. As expected, rosiglitazone, a PPAR-*γ* agonist, activated PPAR-*γ* (Figure 4). However, the crude extract of *P. cocos*, dehydrotrametenolic acid, and pachymic acid did not activate PPAR-*γ* (Figure 4). All the data suggest that *P. cocos* and triterpenes reduce blood glucose level via an enhancement of insulin sensitivity independent of PPAR-*γ* (Figure 5).

## 4. Discussion

*P. cocos* is an edible fungus and is often used in various Chinese herbal medicines [13]. One of its claimed benefits is to treat diabetes; however, the actual anti-diabetic effects, active constituent(s), and mode of action of* P. cocos* have not been extensively studied. Here, we demonstrated, for the first time, that *P. cocos* shows an anti-diabetic effect in db/db mice as evidenced by its glucose-lowering activity (Figure 2). Moreover, we showed, for the first time, that dehydrotumulosic acid is one of the most effective compounds present in the crude extract of *P. cocos* (Figure 2). Dehydrotrametenolic acid and, probably, pachymic acid were reported to be active compounds of *P. cocos* for glucose reduction in db/db mice [20]. Our data confirmed that dehydrotrametenolic acid and pachymic acid have lower glucose-reducing activities than dehydrotumulosic acid (Figure 2(d)). The anti-diabetic mechanism of *P. cocos* and its triterpenes are likely due to their abilities to sensitize insulin-mediated glucose uptake (Figure 3) [27]. Therefore, our findings provide insights into the mechanism of action of *P. cocos *and its active triterpenes against diabetes.

Lanostane-type triterpenes, pachymic acid, dehydrotrametenolic acid, polyporenic acid C, dehydropachymic acid and 3-O-acetyl-16*α*-hydroxy-dehydrotrametenolic acid, and 3,4-secolanostane-type triterpenes, poricoic acid A, B, and D were identified from *P. cocos* [20]. Among these compounds, dehydrotrametenolic acid at 110 mg/kg body weight/day was confirmed as an active triterpene against hyperglycemia in db/db mice. In this paper, dehydrotumulosic acid was identified from *P. cocos* crude extract using a bioactivity-directed fractionation and isolation strategy (Figures 1 and 2). However, dehydrotrametenolic acid (in chloroform sub-fraction 3) and pachymic acid (in chloroform sub-fraction 2) were not present in the active chloroform sub-fractions 4 and 6 of *P. cocos*. Two reasons may contribute to this contradiction. First, dehydrotrametenolic acid and pachymic acid have lower bioactivities than dehydrotumulosic acid. Second, we found that the percentages of dehydrotumulosic acid, dehydrotrametenolic acid, and pachymic acid in* P. cocos* crude extract were 0.012‰, 0.0022‰ and 0.053‰, respectively. The animal experiments in db/db mice showed that dehydrotumulosic acid and dehydrotrametenolic acid had higher anti-hyperglycemic activity than pachymic acid (Figure 2(d)). Our data suggest that triterpenes are the primary compounds present in the crude extract of *P. cocos* despite their bioactivities with different degrees (Figures 1(a) and 2). Moreover, structures of these 3 compounds were confirmed by a comparison of the NMR, MS, and UV spectroscopy data obtained with published data from [21–23].

Crude extract of *P. cocos* and its triterpenes could reduce blood glucose via increased insulin sensitivity, as evidenced by STZ-treated mice (Figure 3). It is possible that the increase in insulin sensitivity could be partially achieved by modification/activation of the PPAR*-* *γ* pathway. In fact, our data showed that *P. cocos* and its triterpenes did not activate PPAR*-* *γ*, a target of rosiglitazone (Figure 4). The mechanism by which crude extract of *P. cocos* and its triterpenes exert insulin sensitizer activity is PPAR*-* *γ* independent. Biguanides (e.g., metformin) are another type of insulin sensitizer, which activate 5′ AMP-activated protein kinase. Whether or not *P. cocos* and its triterpenes affect 5′ AMP-activated protein kinase needs to be further studied [28]. 

Overall, db/db mice and STZ-treated mice experiments showed that the crude extract and triterpenes dehydrotumulosic acid and dehydrotrametenolic acid of *P. cocos* significantly enhanced the insulin sensitivity and, consequently, lowered the blood glucose level in a diabetic mouse model. This enhancement is independent of PPAR*-* *γ* activation.
